# Impact of Neighborhood Socioeconomic Conditions on the Risk of Stroke in Japan

**DOI:** 10.2188/jea.JE20140117

**Published:** 2015-03-05

**Authors:** Kaori Honjo, Hiroyasu Iso, Tomoki Nakaya, Tomoya Hanibuchi, Ai Ikeda, Manami Inoue, Norie Sawada, Shoichiro Tsugane, the Japan Public Health Center-based Prospective Study Group

**Affiliations:** 1 Global Collaboration Center, Osaka University, Osaka, Japan; 1 大阪大学グローバルコラボレーションセンター; 2 Department of Social Medicine, Osaka University Graduate School of Medicine, Osaka, Japan; 2 大阪大学大学院医学研究科 公衆衛生学教室; 3 Department of Geography and Research Institute for Disaster Mitigation of Urban Cultural Heritage, Ritsumeikan University, Kyoto, Japan; 3 立命館大学 文学部 地理学教室; 4 School of International Liberal Studies, Chukyo University, Nagoya, Japan; 4 中京大学 国際教養学部; 5 Department of Public Health, Juntendo University Graduate School of Medicine, Tokyo, Japan; 5 順天堂大学医学部 公衆衛生学教室; 6 Epidemiology and Prevention Group, Research Center for Cancer Prevention and Screening, National Cancer Center, Tokyo, Japan; 6 国立がんセンター がん予防・検診研究センター; 7 Graduate School of Medicine, The University of Tokyo, Tokyo, Japan; 7 東京大学医学系研究科 国際保健政策学教室

**Keywords:** neighborhood, stroke, socioeconomic status, poverty areas, Japan

## Abstract

**Background:**

Neighborhood deprivation has been shown in many studies to be an influential factor in cardiovascular disease risk. However, no previous studies have examined the effect of neighborhood socioeconomic conditions on the risk of stroke in Asian countries.

**Methods:**

This study investigated whether neighborhood deprivation was associated with the risk of stroke and stroke death using data from the Japan Public Health Center-based Prospective Study. We calculated the adjusted hazard ratios of stroke mortality (mean follow-up, 16.4 years) and stroke incidence (mean follow-up, 15.4 years) according to the area deprivation index (ADI) among 90 843 Japanese men and women aged 40–69 years. A Cox proportional-hazard regression model using a shared frailty model was applied.

**Results:**

The adjusted hazard ratios of stroke incidence, in order of increasing deprivation with reference to the least deprived area, were 1.16 (95% CI, 1.04–1.29), 1.12 (95% CI, 1.00–1.26), 1.18 (95% CI, 1.02–1.35), and 1.19 (95% CI, 1.01–1.41), after adjustment for individual socioeconomic conditions. Behavioral and psychosocial factors attenuated the association, but the association remained significant. The associations were explained by adjusting for biological cardiovascular risk factors. No significant association with stroke mortality was identified.

**Conclusions:**

Our results indicate that the neighborhood deprivation level influences stroke incidence in Japan, suggesting that area socioeconomic conditions could be a potential target for public health intervention to reduce the risk of stroke.

## Abstract (ja)

**背景—:**

居住地域の社会経済状況が循環器疾患リスクと関連があることは主に欧米の先行研究により報告されているが、これまでにアジア地域からの報告はない。

**方法—:**

本研究は、居住地域の社会経済状況と脳卒中死亡・罹患リスクの関連について日本の大規模コホートのひとつであるJPHCコホート研究のデータを用いて検証することを目的とした。対象者はJPHC研究に参加した40-69歳の地域住民90,843人。居住地域の社会経済状況を示す指標として、国勢調査などの多数の地域統計指標を合成して推定した近隣居住地域剥奪度(Area Deprivation Index: ADI)を5分位に分類し用いた。平均16.4年間の脳卒中死亡および平均15.4年間の罹患を追跡した。マルチレベル生存分析を用い、年齢、性別、職業、保健所地域、近隣居住地域の人口密度を調整したハザード比を算出した。

**結果―:**

脳卒中罹患リスクにおける居住地域剥奪度の調整ハザード比は、剥奪度最低群（1群：最も裕福）を基準として、1.16 (95%CI, 1.04-1.29) (2群)、1.12 (95%CI, 1.00-1.26) (3群)、1.18 (95%CI, 1.02-1.35) (4群)、1.19 (95%CI, 1.01-1.41) (5群：最も貧しい)であった。脳卒中死亡リスクとの関連は見られなかった。

**結論—:**

本研究の結果、近隣居住地域の社会経済状況とそこに居住する人の脳卒中罹患リスクが関連することを示した。貧しい地域に居住する人は豊かな地域に居住する人と比較して、脳卒中のリスクが低い傾向がみられ、日本における脳卒中予防において地域の社会経済状況を考慮する必要があることが示唆された。

## INTRODUCTION

A wealth of evidence has consistently demonstrated that the level of neighborhood deprivation is an influential factor in the risk of cardiovascular disease.^1^ There is a strong, independent association between living in a socioeconomically disadvantaged neighborhood and risk of coronary heart disease,^2^^,^^3^ total stroke,^4^^–^^7^ and ischemic stroke,^8^^–^^10^ even after adjustment for individual socioeconomic position. However, to our knowledge, there have been no studies examining neighborhood socioeconomic conditions in relation to the risk of stroke among general residents in Asian countries.

Evidence of social inequalities in cardiovascular disease has been accumulating in Japan^11^; several studies have shown differences in mortality, morbidity, and risk factors for cardiovascular disease—stroke in particular—according to indicators of individual socioeconomic conditions.^12^^–^^16^ Regional inequalities in health have also been repeatedly reported. Several ecological studies have indicated that municipal-level socioeconomic conditions exert an influence on the health of the population in Japan: lower municipal-level socioeconomic conditions have had an adverse influence on population health,^17^^–^^19^ health behaviors (eg, smoking, excess alcohol consumption, and physical inactivity),^20^ and body mass index (BMI).^21^ All of those studies were cross-sectional or ecological studies. One exception, a recently-conducted multi-level study, indicated that higher municipal deprivation levels were associated with increased risk of cardiovascular mortality among men.^22^ However, thus far, no prospective studies have been conducted to examine the impact of finer neighborhood-level socioeconomic conditions on individual risk of developing cardiovascular diseases.

In this study, we aimed to investigate whether census-based neighborhood-level socioeconomic conditions are associated with stroke mortality and incidence, using data from a large prospective cohort study carried out in a Japanese non-metropolitan setting.

## METHODS

### Study cohort

We used data from cohorts 1 and 2 of the Japan Public Health Center-based Prospective Study (JPHC Study), a large population-based prospective study of 140 420 men and women aged 40–69 years.^23^ Two Public Health Center (PHC) areas in metropolitan Tokyo and Osaka were excluded from the present analysis (*n* = 23 524) because no data on stroke incidence were available. Of the remaining 116 896 participants, 9 were excluded as ineligible (7 were non-Japanese and 2 had moved prior to the start of the study).

The baseline self-administered questionnaire was distributed to all registered participants in 1990 (for cohort 1) and 1993 (for cohort 2), and the overall response rate was 81.6%. Of the 95 405 participants, we excluded 32 ineligible subjects and 3650 with a history of cancer or cardiovascular diseases. The remaining 91 723 participants were considered eligible for inclusion in this analysis.

### Measurement

To examine the effect of neighborhood deprivation on stroke mortality and incidence, this study used area deprivation data at the level of the *chocho-aza* (CA) unit, the smallest administrative unit in Japan, which is roughly comparable to a European parish or a U.S. block-group-size neighborhood. We excluded four subjects whose addresses could not be geocoded. We calculated the CA-level area deprivation index (ADI), which is a composite indicator consisting of the weighted sum of a number of census-based variables. The ADI was derived and has been described in detail by Nakaya.^24^^,^^25^ In brief, we constructed an ADI for each of 695 CAs. The ADI was calculated using deprivation-related census-based variables (eg, the proportions of elderly couple households, elderly single households, single-mother households, sales and service workers, agricultural workers, blue-collar workers, and non-employed persons) in the 1995 population census in each area unit. The ADI was tested against ecological datasets of all-cause and various cancer mortalities at the municipal level across Japan and showed consistent positive relationships with most mortality indices.^24^ In addition, we confirmed that higher ADI was associated with increased risk of all-cause death using the same method.^25^

We considered occupation as an indicator of individual socioeconomic conditions. Occupation was categorized into nine groups: professional, management, office work, sales/service, transportation/communication industries and not classifiable, manual labor, agriculture/forestry/fishery, non-working, and information missing.

Age, gender, PHC district, and population density at the CA level were considered as confounding factors, and biological cardiovascular risk factors (hypertension, diabetes or hyperlipidemia, and overweight) and behavioral and psychosocial factors (perceived psychological stress, marital status, smoking, alcohol intake, and physical activity) were considered as hypothesized mediating factors.

### Study participants

Of the 91 723 eligible participants in this cohort study, we further excluded 880 subjects because no census information was provided by the statistical bureau for their CAs, or their CAs had a small number of households (less than 25). The remaining 90 843 men and women were included in the analysis. The median area was 2.39 km^2^ (range, 0.02–56.28 km^2^), median population was 1234 (range, 68–9330), and median number of households was 363 (range, 25–3254). The study was approved by the human ethics review committee of the National Cancer Center.

### Confirmation of stroke incidence and mortality

A total of 78 hospitals were registered within the administrative districts of the JPHC cohort. All were major hospitals capable of treating patients with stroke. Physicians blinded to the patients’ lifestyle data reviewed the medical records at each hospital. Strokes were confirmed according to the criteria of the National Survey of Stroke,^26^ which requires the presence of a focal neurological deficit of sudden or rapid onset lasting at least 24 hours or until death. All of the registered hospitals were major hospitals with admission facilities for acute cardiovascular events and were equipped with a computed tomographic scanning and/or magnetic resonance imaging apparatus. In addition to annual surveillance, we conducted another search for unidentified events. When subjects reported a history of nonfatal stroke on the 5- and 10-year follow-up questionnaire and had not been registered as stroke cases, we asked by letter or telephone about the onset of stroke and for permission to review medical records. Thus, we assume that most of the acute stroke cases were captured.

All death certificates were forwarded centrally to the Ministry of Health, Labour and Welfare, and coded for the National Vital Statistics. Registration of death is required by the Family Registration Law and is believed to be complete in Japan. The underlying causes of deaths were defined according to the International Classification of Diseases, 10th Revision, as deaths from stroke (codes I60 to I69).

Stroke deaths and events were included in the analyses if they occurred after the date of return of the baseline questionnaire and before January 1, 2010 (for cohort 1), and January 1, 2008 (for cohort 2).

### Statistical analysis

The outcomes for this study were defined as newly occurring stroke incidence and deaths during the study period. Person-years were counted from the date of the return of the baseline survey until one of the following end points. For the analysis of stroke incidence, person-years were censored at the date of disease diagnosis, the date of emigration from the study area, the date of death, or the end of the study period (December 31, 2009, for cohort 1 and December 31, 2007, for cohort 2), whichever came first. For the analysis of stroke deaths, person-years were censored at the date of emigration from the study area, the date of death, or the end of the study period (December 31, 2010, for cohorts 1 and 2), whichever came first. For persons who were lost to follow-up, the last confirmed date of their presence in the study area was used as the date of censoring.

We estimated adjusted hazard ratios (HRs) and 95% confidence intervals (CIs) of stroke incidence and mortality by applying a Cox proportional-hazard regression model using a shared frailty model that allows individuals to be nested within neighborhoods and the intercept to vary between neighborhoods.^27^ We assumed two area levels: broad regional differences in living situations (adjusted for by including dummy variables of PHC district for each person) and the CA level (covering possible clustering tendencies within the same neighborhood according to the random intercept of the shared frailty function). Model fitting procedures were carried out using the PHREG command with RANDOM statement by SAS ver. 9.3 (SAS Institute Inc., Cary, NC, USA).

The dummy variables of PHC district and the random effect were included for all of the fitted models to calculate adjusted HRs and 95% CIs (Model 1). Further adjustment was made for occupation category considered as an individual socioeconomic indicator, as well as age, gender, and population density considered as potential confounding variables (Model 2). To examine the mediating mechanism, social and behavioral factors (marital status, perceived psychological stress, smoking behavior, frequency and amount of alcohol intake, and frequency of physical activity during leisure; Model 3) and biological cardiovascular risk factors (overweight, medical history of hypertension, diabetes, or hyperlipidemia; Model 4) were inserted into the model. All analyses were conducted with SAS statistical package version 9.3 (SAS Institute Inc.).

## RESULTS

Table 1 lists the characteristics of the study population and their associations with ADI level. Participants residing in the most deprived neighborhoods were more likely to be older, to have agriculture/forestry/fishery jobs, to be overweight, and to live alone, and were less likely to smoke, to have a medical history of hypertension, and to perceive they had psychological stress, compared with those in less deprived neighborhoods. We identified 1147 stroke deaths during a mean follow-up period of 16.4 years, while 4410 stroke events were documented during a mean follow-up period of 15.4 years.

**Table 1.** Characteristics of study subjects

	Total	Area Deprivation Index					*P*-value for difference^a^

		0 (Least deprived)	1	2	3	4 (Most deprived)	
*n*	90 843	18 159	17 992	18 230	18 282	18 180	
**Mean (SD)**							
Age, years	50.3 (7.6)	49.8 (7.9)	49.9 (7.4)	49.9 (7.4)	50.5 (7.5)	51.3 (7.7)	<0.0001
BMI, kg/m^2^	23.5 (3.1)	23.2 (2.9)	23.2 (2.9)	23.3 (3.0)	23.8 (3.1)	24.0 (3.2)	<0.0001

**Frequency (%)**							
Gender							
Men	43 337 (48)	8827 (49)	8701 (48)	8599 (47)	8583 (47)	8627 (47)	0.003
Occupation							
Professional	3587 (4)	747 (4)	738 (4)	791 (4)	667 (4)	644 (4)	<0.0001
Manager	2898 (3)	915 (5)	640 (4)	555 (3)	435 (2)	353 (2)	
Clerk	7227 (8)	1654 (9)	1640 (9)	1602 (9)	1242 (7)	1089 (6)	
Agriculture/Forestry/Fishery	21 790 (24)	3358 (18)	3418 (19)	3817 (21)	5335 (29)	5862 (32)	
Service/Sales	7187 (8)	1717 (9)	1405 (8)	1440 (8)	1452 (8)	1173 (6)	
Manual job	10 573 (12)	2022 (11)	2181 (12)	2451 (13)	2083 (11)	1836 (10)	
No job	18 596 (20)	3802 (21)	4379 (24)	4058 (22)	3395 (19)	2962 (16)	
Security/transportation/ communication/others	17 759 (20)	3742 (21)	3350 (19)	3208 (18)	3428 (19)	4031 (22)	
Missing	1226 (1)	202 (1)	241 (1)	308 (2)	245 (1)	230 (1)	
Marital status							
Married	73 356	15 335 (84)	14 809 (82)	14 666 (80)	14 226 (78)	14 320 (79)	<0.0001
Unmarried	16 761	2656 (16)	3047 (18)	3399 (19)	3893 (21)	3766 (21)	
Missing	726	168 (1)	136 (1)	165 (1)	163 (1)	94 (1)	
Perceived psychological stress							
Low	14 195 (16)	2515 (14)	2449 (14)	2626 (14)	3091 (17)	3514 (19)	<0.0001
Medium	57 233 (63)	11 419 (63)	11 246 (63)	11 389 (63)	141 661 (64)	11 518 (63)	
High	17 923 (20)	3861 (21)	3954 (22)	3867 (21)	3270 (18)	2971 (16)	
Missing	1492 (2)	364 (2)	343 (2)	348 (2)	260 (1)	177 (1)	
Smoking							
Never smoker	54 344 (60)	10 245 (56)	10 260 (57)	10 838 (59)	11 415 (62)	11 586 (64)	<0.0001
Quitter	10 507 (12)	2231 (12)	2117 (12)	2044 (11)	1957 (11)	2158 (12)	
Current smoker	25 563 (28)	5548 (31)	5507 (31)	5287 (29)	4852 (27)	4369 (24)	
Missing	429 (0.5)	135 (0.7)	108 (0.6)	61 (0.3)	58 (0.3)	67 (0.4)	
Ethanol intake							
No	46 815 (52)	8708 (48)	8743 (49)	8902 (48)	9988 (54)	10 474 (57)	<0.0001
Occasional drinker	8628 (10)	1583 (9)	1681 (9)	1762 (10)	1848 (10)	1754 (10)	
149 g and less per week	10 337 (11)	2322 (13)	2122 (12)	2341 (13)	1889 (10)	1663 (9)	
150–299 g per week	8494 (10)	1866 (10)	1836 (10)	1817 (10)	1613 (9)	1362 (8)	
300–449 g per week	7331 (8)	1635 (9)	1656 (9)	1421 (8)	982 (5)	708 (4)	
450 g and more per week	7331 (8)	1505 (8)	1501 (8)	1465 (8)	1431 (8)	1429 (8)	
Missing	2836 (3)	540 (3)	453 (3)	522 (3)	531 (3)	790 (4)	
Physical activity at leisure time							
No	29 490 (32)	4307 (24)	6365 (35)	6429 (35)	6562 (36)	5830 (32)	<0.0001
1–3 times per month	39 268 (43)	9030 (50)	7311 (41)	7512 (41)	7266 (40)	8149 (45)	
1–2 times per week	8398 (9)	1887 (10)	1768 (10)	1618 (9)	1536 (8)	1589 (9)	
3 times and more per week	12 492 (14)	2651 (15)	2265 (13)	2412 (13)	2702 (15)	2462 (14)	
Missing	1195 (1)	287 (2)	283 (2)	259 (1)	216 (1)	150 (1)	
Hypertension							
Yes	16 575 (18)	3539 (19)	3388 (19)	3306 (18)	3259 (18)	3083 (17)	<0.0001
Missing	141 (0.2)	33 (0.2)	47 (0.3)	21 (0.1)	17 (0.1)	23 (0.1)	
Diabetes mellitus							
Yes	4210 (5)	853 (5)	840 (5)	867 (5)	883 (5)	767 (4)	0.0001
Missing	141 (0.2)	33 (0.2)	47 (0.3)	22 (0.1)	16 (0.1)	23 (0.1)	
Hyperlipidemia							
Yes	1530 (2)	423 (2)	347 (2)	326 (2)	251 (1)	183 (1)	<0.0001
Overweight (BMI ≥25)							
Yes	25 630 (28)	4394 (24)	4422 (25)	4851 (27)	5717 (31)	6246 (34)	<0.0001
Missing	1029 (1)	175 (1)	172 (1)	233 (1)	232 (1)	217 (1)	

BMI, body mass index; SD, standard deviation.

^a^ANOVA test or Chi-square test were used.

Table 2 shows the adjusted HRs and 95% CIs of stroke mortality and incidence according to ADI level. A significant association was identified between neighborhood deprivation level and the risk of developing stroke. The adjusted HRs for risk of developing stroke, in order of increasing deprivation with reference to the least deprived area, were 1.16 (95% CI, 1.04–1.29), 1.12 (95% CI, 1.00–1.26), 1.18 (95% CI, 1.02–1.35), and 1.19 (95% CI, 1.01–1.41), after adjustment for individual socioeconomic conditions. Behavioral and psychosocial factors attenuated the association, but the association remained significant. The association was explained by adjusting for biological cardiovascular risk factors. On the other hand, no association was identified between neighborhood deprivation level and the risk of stroke death.

**Table 2.** Multivariate hazard ratios and 95% confidence intervals of stroke incidence and mortality according to area deprivation level among 90 843 men and women

	Area Deprivation Index					Trend *P*-value	Log- likelihood	Random effects (SD)

	0 (Least deprived)	1	2	3	4 (Most deprived)			
*n*	18 159	17 992	18 230	18 282	18 180			
Death								
Person-years	288 735	300 655	304 956	302 767	296 639			
Number of cases	202	225	242	226	252			
Model 0							−12 858	0.08 (0.03)
Model 1	1.00	1.17 (0.95, 1.43)	1.32 (1.06, 1.65)	1.36 (1.05, 1.75)	1.79 (1.34, 2.39)	0.004	−12 817	0.024 (0.021)
Model 2	1.00	1.00 (0.82, 1.22)	1.04 (0.84, 1.28)	0.99 (0.76, 1.27)	1.02 (0.76, 1.38)	0.76	−12 495	0.001 (0.019)
Model 3	1.00	1.00 (0.82, 1.22)	1.04 (0.84, 1.29)	0.99 (0.76, 1.28)	1.02 (0.76, 1.37)	0.75	−12 448	0.001 (0.019)
Model 4	1.00	1.00 (0.82, 1.22)	1.03 (0.83, 1.29)	0.99 (0.77, 1.28)	1.02 (0.76, 1.38)	0.75	−12 450	0.002 (0.019)
Incidence								
Person-years	273 578	282 021	283 995	282 766	274 064			
Number of cases	735	911	879	959	926			
Model 0							−49 061	0.049 (0.010)
Model 1	1.00	1.20 (1.06, 1.35)	1.14 (1.00, 1.30)	1.24 (1.07, 1.44)	1.32 (1.12, 1.56)	0.01	−49 027	0.029 (0.009)
Model 2	1.00	1.16 (1.04, 1.29)	1.12 (1.00, 1.26)	1.18 (1.02, 1.35)	1.19 (1.01, 1.41)	0.13	−48 209	0.009 (0.006)
Model 3	1.00	1.13 (1.02, 1.26)	1.09 (0.97, 1.23)	1.14 (1.00, 1.31)	1.13 (0.96, 1.33)	0.14	−48 094	0.008 (0.006)
Model 4	1.00	1.06 (0.96, 1.18)	1.02 (0.91, 1.14)	1.06 (0.93, 1.22)	1.05 (0.90, 1.23)	0.13	−47 984	0.003 (0.005)

SD, standard deviation.

Model 0 = Null model.

Model 1 = Area Deprivation Index (ADI) and PHC district were in the model.

Model 2 = Model 1 + age, gender, occupation and population density.

Model 3 = Model 2 + Behavioral and psychosocial factors (smoking, ethanol intake, physical activity, perceived psychological stress, marital status).

Model 4 = Model 2 + CVD biological risk factors (overweight, medical history of hypertension/diabetes/hyperlipidemia).

## DISCUSSION

In this cohort, we observed that neighborhood deprivation level was associated with total stroke incidence after adjustment for individual socioeconomic indicators, and living in more deprived neighborhoods increased the risk of developing stroke regardless of individual socioeconomic conditions. The association remained after adjustment for individual socioeconomic indicators, which suggests that the neighborhood deprivation level has an impact on disparities in stroke incidence in Japanese society. Our results suggest that neighborhood socioeconomic conditions could be a potential target for public health interventions to reduce the risk of stroke in certain areas.

Our results were consistent with results of previous studies in Western societies that found that residing in disadvantaged neighborhoods is associated with increased risk of stroke.^1^^,^^8^^–^^10^ Neighborhood socioeconomic conditions are assumed to be associated with the physical and social environments of neighborhoods, which impact cardiovascular health mediated through cardiovascular risk factors. For example, there is a higher prevalence of health-damaging behaviors and reduced use of healthcare resources in disadvantaged neighborhoods.^8^^,^^28^^–^^32^

Our mediation analysis showed some attenuation of HRs after adjusting for behavioral and psychosocial risk factors at an individual level, but the association between ADI and stroke risk remained significant. However, residual confounding from other important risk factors, such as mental health (eg, depression) or measurement errors in our variables, could influence the association. Biological cardiovascular risk factors explained the association between ADI and stroke risk, which suggests that the impact of neighborhood deprivation level on stroke risk could be a result of unequal distribution of biological cardiovascular risk factors. Further detailed research is needed to examine how the neighborhood socioeconomic factors influence stroke incidence in Japanese society.

We identified no association of ADI with risk of death from stroke, which was inconsistent with studies in Western societies.^33^ One possible explanation for the discrepancy could be an inappropriate level of analysis of stroke mortality. In terms of stroke death, secondary prevention, such as acute treatment and medical services, could be crucial. In Japan, provision of medical services has been implemented over large areas, such as medical districts. Thus, our level of analysis may be inappropriate to detect an area effect on stroke mortality.

Several limitations of this study warrant discussion. First, the generalizability of our study results could be limited. The data included nine public health districts in a non-metropolitan setting, so we should be cautious in interpreting our results, especially in metropolitan areas. Second, there was a 5-year gap between census data used for the ADI and the baseline data. However, the area socioeconomic characteristics were not likely to change dramatically in Japan, especially in non-metropolitan areas, so we assumed that the 5-year gap did not have a major effect on our results. Third, we used CA units as an indirect proxy for neighborhood, but we cannot negate the possibility that they may not reflect meaningful neighborhood boundaries relevant to cardiovascular disease. Fourth, we adjusted for individual-level socioeconomic conditions using occupation only; however, occupation may not be a strong enough indicator for individual socioeconomic conditions. Unfortunately, we did not have sufficient information on the participants’ socioeconomic status to fully adjust for individual socioeconomic conditions.

In summary, we found that residing in a deprived neighborhood influences the risk of stroke in Japan. The results indicate that neighborhood deprivation contributes to disparities in the risk of stroke in Japanese society, and suggest that related socioeconomic factors could be a potential target for public health intervention to reduce stroke incidence. Future research is necessary to further investigate how neighborhood physical and social factors influence cardiovascular risk.

## ONLINE ONLY MATERIAL

Abstract in Japanese.
