# Supplementary material for: Impact of Neighborhood Socioeconomic Conditions on the Risk of Stroke in Japan
Source: J Epidemiol. 2015 Mar 5;25(3):254–60. doi: 10.2188/jea.JE20140117 (PMC4341003; doi:10.2188/jea.JE20140117)
Supplement: Abstract in Japanese. [file je-25-254-s001.pdf]

# 日本における近隣居住地域の社会経済状況と脳卒中リスク

本庄かおり [1]　磯　博康 [2]　中谷友樹 [3]　埴淵知哉 [4]　池田　愛 [5]　井上真奈美 [6,7]
澤田典絵 [6]　津金昌一郎 [6]　JPHC 研究グループ

[1] 大阪大学グローバルコラボレーションセンター
[2] 大阪大学大学院医学研究科　公衆衛生学教室
[3] 立命館大学 文学部　地理学教室
[4] 中京大学　国際教養学部
[5] 順天堂大学医学部　公衆衛生学教室
[6] 国立がんセンター　がん予防・検診研究センター
[7] 東京大学医学系研究科　国際保健政策学教室

**抄録**
背景—居住地域の社会経済状況が循環器疾患リスクと関連があることは主に欧米の先行研究により報告されているが、これまでにアジア地域からの報告はない。

方法—本研究は、居住地域の社会経済状況と脳卒中死亡・罹患リスクの関連について日本の大規模コホートのひとつである JPHC コホート研究のデータを用いて検証することを目的とした。対象者は JPHC 研究に参加した 40-69 歳の地域住民 90,843 人。居住地域の社会経済状況を示す指標として、国勢調査などの多数の地域統計指標を合成して推定した近隣居住地域剥奪度(Area Deprivation Index: ADI)を 5 分位に分類し用いた。平均 16.4 年間の脳卒中死亡および平均 15.4 年間の罹患を追跡した。マルチレベル生存分析を用い、年齢、性別、職業、保健所地域、近隣居住地域の人口密度を調整したハザード比を算出した。

結果—脳卒中罹患リスクにおける居住地域剥奪度の調整ハザード比は、剥奪度最低群（1 群：最も裕福）を基準として、1.16 (95%CI, 1.04-1.29) (2 群)、 1.12 (95%CI, 1.00-1.26) (3 群)、 1.18 (95%CI, 1.02-1.35) (4 群)、 1.19 (95%CI, 1.01-1.41) (5 群：最も貧しい)であった。脳卒中死亡リスクとの関連は見られなかった。

結論—本研究の結果、近隣居住地域の社会経済状況とそこに居住する人の脳卒中罹患リスクが関連することを示した。貧しい地域に居住する人は豊かな地域に居住する人と比較して、脳卒中のリスクが低い傾向がみられ、日本における脳卒中予防において地域の社会経済状況を考慮する必要があることが示唆された。

**キーワード：** 近隣地区 • 脳卒中• 社会経済状況 •貧困• 日本
